# Identification and characterization of *Arabidopsis* AtNUDX9 as a GDP-d-mannose pyrophosphohydrolase: its involvement in root growth inhibition in response to ammonium

**DOI:** 10.1093/jxb/erv281

**Published:** 2015-06-06

**Authors:** Hiroyuki Tanaka, Takanori Maruta, Takahisa Ogawa, Noriaki Tanabe, Masahiro Tamoi, Kazuya Yoshimura, Shigeru Shigeoka

**Affiliations:** ^1^Department of Advanced Bioscience, Faculty of Agriculture, Kinki University, 3327-204 Nakamachi, Nara 631–8505, Japan; ^2^Department of Life Science and Biotechnology, Faculty of Life and Environmental Science, Shimane University, 1060 Nishikawatsu, Matsue, Shimane 690–8504, Japan; ^3^Department of Food and Nutritional Science, College of Bioscience and Biotechnology, Chubu University,1200 Matsumoto-cho, Kasugai, Aichi, 487–8501Japan

**Keywords:** Ammonium response, *Arabidopsis*, GDP-d-mannose pyrophosphohydrolase, l-ascorbic acid, protein N-glycosylation, Nudix hydrolase.

## Abstract

GDP-d-mannose (GDP-d-Man) is an important intermediate in ascorbic acid (AsA) synthesis, cell wall synthesis, protein N-glycosylation, and glycosylphosphatidylinositol-anchoring in plants. Thus, the modulation of intracellular levels of GDP-d-Man could be important for maintaining various cellular processes. Here an *Arabidopsis* GDP-d-Man pyrophosphohydrolase, AtNUDX9 (AtNUDT9; At3g46200), which hydrolysed GDP-d-Man to GMP and mannose 1-phosphate, was identified. The *K*_m_ and *V*_max_ values for GDP-d-Man of AtNUDX9 were 376±24 μM and 1.61±0.15 μmol min^–1^ mg^–1^ protein, respectively. Among various tissues, the expression levels of AtNUDX9 and the total activity of GDP-d-Man pyrophosphohydrolase were the highest in the roots. The GDP-d-Man pyrophosphohydrolase activity was increased in the root of plants grown in the presence of ammonium. No difference was observed in the levels of AsA in the leaf and root tissues of the wild-type and knockout-*nudx9* (KO-*nudx9*) plants, whereas a marked increase in N-glycoprotein levels and enhanced growth were detected in the roots of KO-*nudx9* plants in the presence of ammonium. These results suggest that AtNUDX9 is involved in the regulation of GDP-d-Man levels affecting ammonium sensitivity via modulation of protein N-glycosylation in the roots.

## Highlight

AtNUDX9, a GDP-d-Man pyrophosphohydrolase in *Arabidopsis*, is involved in the regulation of GDP-d-Man levels affecting ammonium sensitivity via modulation of protein N-glycosylation in the roots.

## Introduction

GDP-d-mannose (GDP-d-Man) is an activated sugar nucleotide that is required for many aspects of plant metabolism. This compound is necessary for the biosynthesis of ascorbic acid (AsA), an essential antioxidant in plants, and some different structural carbohydrates for the synthesis of cell wall polysaccharides, as a precursor (Bonin *et al.*, 1997; Conklin *et al.*, 1999) (see Supplementary Fig. S1 at *JXB* online). GDP-d-Man is also essential for post-translational modifications such as protein N-glycosylation and glycosylphosphatidylinositol (GPI)-anchoring (Qian *et al.*, 2007; Jadid *et al.*, 2011) and for the synthesis of GDP-l-fucose, a constituent of many structural polysaccharides and glycoproteins (Reiter and Vauzin, 2001). Three enzymes located in the cytosol, phosphomannose isomerase (PMI), phosphomannose mutase (PMM), and GDP-d-mannose pyrophosphorylase (GMP/VTC1), have been linked to the biosynthesis of GDP-d-Man (see Supplementary Fig. S1 at *JXB* online). Of these, VTC1 catalyses the reversible pyrophosphorylation: d-Man 1-phosphate+GTP↔GDP-d-Man+pyrophosphate (Conklin *et al.*, 1999). *Arabidopsis vtc1-1* mutants, which accumulate low levels of AsA (approximately 35% that of wild-type plants), show pleiotropic phenotypes, such as altered defence in response to oxidative stress and hormone homeostasis, and inhibited growth and development (Conklin *et al.*, 1996, 2000; Lukowitz *et al.*, 2001; Pastori *et al.*, 2003). On the other hand, a mutation in the gene encoding enzymes downstream of VTC1 in the AsA biosynthetic pathway, l-galactose-1-phosphate phosphatase (GPP/VTC4), whose products are only used to synthesize AsA (see Supplementary Fig. S1 at *JXB* online), hardly showed such pleiotropic phenotypes (Qin *et al.*, 2008). Thus, it appears that some of these pleiotropic phenotypes of *vtc1* cannot be directly attributed to an AsA deficiency, suggesting the importance of maintaining appropriate levels of GDP-d-Man in cells.

A relationship has recently been reported between the metabolism of GDP-d-Man and sensitivity to ammonium (${\text{NH}}_{\text{4}}{}^{+}$ ), a major nitrogen source for plants. A previous study demonstrated that root growth by *vtc1-1* mutants was stunted in the presence of ${\text{NH}}_{\text{4}}{}^{+}$ , whereas they developed roots similar to those of wild-type plants in the absence of ${\text{NH}}_{\text{4}}{}^{+}$ (Barth *et al.*, 2010). The *vtc1-1* mutants were also found to have N-glycosylation defects, enhanced programmed cell death, and some cell-cycle defects in the presence of NH_4_^+^ (Qin *et al.*, 2008; Kempinski *et al.*, 2011). Root elongation was previously shown to be sensitive to ${\text{NH}}_{\text{4}}{}^{+}$ in the mutants of DPMS1 (*dpms1-1*), which acts downstream of VTC1 and mediates the biosynthesis of dolichol phosphate mannose (Dol-phosphate-Man), required for the synthesis of N-glycoproteins and GPI-anchored proteins (Jadid *et al.*, 2011). These previous findings suggested not only that root growth in the presence of ${\text{NH}}_{\text{4}}{}^{+}$ was regulated by protein N-glycosylation, but also that subcellular levels of GDP-d-Man affected various cellular processes possibly via the modulation of cell-wall synthesis, protein N-glycosylation, and GPI-anchoring. However, it currently remains unclear how GDP-d-Man levels are regulated.

Nudix hydrolases are distributed in all living organisms and constitute a large family of proteins that share a highly conserved amino acid sequence, G*X*_5_E*X*_7_REU*X*EE*X*GU, in which U is an aliphatic, hydrophobic residue, although several examples exist with altered consensus sequences (Bessman *et al.*, 1996; McLennan, 2006; Xu *et al.*, 2006). Nudix hydrolases play protective, regulatory, and signalling roles in various metabolic pathways by hydrolysing a wide variety of substrates that contain a nucleoside diphosphate linked to some other moiety, X, such as oxidized nucleotides, dinucleoside polyphosphates, nucleotide sugars, coenzymes, and capped RNAs (Bessman *et al.*, 1996; McLennan, 2006; Kraszewska, 2008, Yoshimura and Shigeoka, 2015). *Arabidopsis* has 28 genes (*AtNUDX1–27* and *AtDCP2*) that encode the Nudix hydrolase homologues distributed to the cytosol and organelles such as chloroplasts and mitochondria (Ogawa *et al.*, 2005, 2008). Although Nudix hydrolases in *Arabidopsis* were initially named as ‘AtNUDT’, we renamed them ‘AtNUDX’ because the *Arabidopsis* gene was not numbered with reference to the closest orthologue of human gene, although both genes were named as NUDT (Yoshimura and Shigeoka, 2015). Various AtNUDXs have been shown to have pyrophosphohydrolase activities with a wide range of substrate specificities; 8-oxo-7,8-dihydro-2′-(deoxy) guanosine 5′-triphosphate (AtNUDX1), ADP-d-ribose (AtNUDX2 and 7), ADP-d-glucose (AtNUDX14), NAD(P)H (AtNUDX6, 7, and 19), coenzyme A (AtNUDX11 and 15), thiamine diphosphate (AtNUDX20), FAD (AtNUDX23), guanosine-3′,5′-tetraphosphate (AtNUDX26), long-chain diadenosine polyphosphates (Ap_n_A)(AtNUDX13, 25, 26, and 27), and mRNA caps (AtDCP2) (Ogawa *et al.*, 2005, 2008, 2009; Munoz *et al.*, 2006; Yoshimura *et al.*, 2007; Gunawardana *et al.*, 2008; Ishikawa *et al.*, 2009, 2010a, *b*; Ito *et al.*, 2012a, b; Maruta *et al.*, 2012; Goyer *et al.*, 2013). However, numerous AtNUDXs (AtNUDX3–5, 8, 9, 12, 16–18, 21, 22, and 24) do not exhibit this activity toward any substrates analysed previously (Ogawa *et al.*, 2005, 2009). GDP-d-Man was identified as a substrate for the Nudix hydrolases, Orf1.9 and Orf191, in *Escherichia coli* (Frick *et al.*, 1995; Xu *et al.*, 2006), suggesting that these enzymes had the potential to be involved in the metabolism of GDP-d-Man.

In the present study, the cytosolic GDP-d-Man pyrophosphohydrolase (AtNUDX9; At3g46200) was identified in *Arabidopsis* plants. The enzymatic properties and tissue-specific expression of AtNUDX9 were investigated as well as the ${\text{NH}}_{\text{4}}{}^{+}$ sensitivity of *AtNUDX9*-disrupted plants. In addition, the levels of AsA and glycoprotein were analysed in the disrupted plants. The present results indicated that AtNUDX9 is involved in the metabolism of GDP-d-Man through the hydrolysis of GDP-d-Man, which then modulates ${\text{NH}}_{\text{4}}{}^{+}$ responses.

## Materials and methods

### Expression and purification of recombinant AtNUDX proteins

The recombinant forms of cytosolic AtNUDXs (AtNUDX1–11 and 25) were produced using *E. coli* strain BL21 (DE3) pLysS cells transformed with pET16b/AtNUDX1–3 and 5–7, pCold II/AtNUDX4, 9–11, and 25, and pCold TF/AtNUDX8 following previously described methods (Ogawa *et al.*, 2005). The respective proteins were purified from the extracts using a His Trap HP column (GE Healthcare, Little Chalfont, UK). Protein contents were determined by the Bradford method, using bovine serum albumin as a standard (Bradford, 1976). The molecular masses of the recombinant AtNUDX proteins were consistent with the predicted values calculated from the amino acid sequence of the mature protein plus the hexahistidine-tag (see Supplementary Fig. S2 at *JXB* online).

### Enzyme assay

GDP-d-Man and GDP-l-fucose were purchased from YAMASA CORPORATION (Chiba, Japan). GDP-d-glucose was purchased from Sigma (St Louis, MO, USA). The hydrolytic activities of the recombinant forms of AtNUDXs toward GDP-d-Man, GDP-d-glucose, and GDP-l-fucose were assayed according to a previously described method (Ogawa *et al.*, 2005). The reaction mixture (60 μl) containing 50mM TRIS–HCl (pH 8.0), 5mM MgCl_2_, 100 μM substrate, and 1.0 μg of the purified recombinant protein, was incubated at 37 °C for 10min. In the assay for *K*_m_, GDP-d-Man was added at 50 μM to 2mM to the reaction. In the assay for divalent cation-dependency, Mg^2+^ was substituted with Cu^2+^, Zn^2+^, Ca^2+^, or Mn^2+^ (1mM or 5mM each). The reaction was terminated by adding 10 μl of 100mM EDTA. The mixture was then analysed by HPLC using a COSMOSIL C18 column (4.6×250mm, Nacalai Tesque, Kyoto, Japan) at a flow rate of 0.6ml min^–1^ for the mobile phase buffer, which contained 73mM KH_2_PO_4_, 5mM tetrabutylammonium dihydrogenphosphate, and 12.5% methanol. The substrates (GDP-d-Man, GDP-d-glucose, and GDP-l-fucose) and reaction product (GMP) were detected by their UV absorbance at 260nm. Blanks without either enzyme or divalent cation were run in parallel.

Due to interference of GDP-d-Man detection by UV absorbance at 260nm by contaminants in the plant extract, GDP-d-Man pyrophosphohydrolase activities in crude extracts from *Arabidopsis* seedlings were determined by detecting d-Man 1- phosphate using high performance anion exchange chromatography coupled with the pulsed amperometric detection (HPAEC-PAD) system using the ICS-3000 ion chromatography system (Dionex, Sunnyvale, CA). The leaves (0.2g) of *Arabidopsis* plants were homogenized with 0.5ml of 100mM TRIS–HCl (pH 8.0) containing 20% glycerol. After centrifugation (20 000×*g*) for 20min at 4 °C, the supernatant was used to analyse enzymatic activity. The reaction mixture (10 μl) was automatically injected onto the column of CarboPac PA1 guard (2×50mm) and CarboPac PA1 (2×250mm), and eluted with the NaOH/sodium acetate gradient. The sodium acetate gradient was increased from 0mM to 200mM between 16min and 24min with a flow rate set at 0.25ml min^–1^. Detection of the reaction product (d-Man 1-phosphate) was achieved by a pulsed amperometric cell using an electrochemical detector equipped with a working gold electrode and combined pH-Ag/AgCl reference electrode.

### Semi-quantitative RT-PCR analysis

Total RNA extracted from various tissues of 5-week-old *Arabidopsis* plants, including rosette leaves, stems, cauline leaves, inflorescences, and roots, was purified with the QuickGene RNA cultured cell Kit S (KURABO, Osaka, Japan), and then treated with DNase I to eliminate any DNA contamination (Takara, Shiga, Japan). First-strand cDNA was synthesized using ReverTra Ace (Toyobo, Osaka, Japan) with an oligo(dT) primer. cDNAs encoding *AtNUDX9* and *Actin8* were semi-quantitatively amplified by PCR using the following primer sets; AtNUDX9-F (5′-GTGTTCGAGCTTCTTCCATGGCG-3′), AtNUDX9-R (5′-CCCCCAGGGAATACATAGTGTCC-3′), Actin8-F (5′-GAGATCCACATCTGCTGG-3′), and Actin8-R (5′-GCTGAGAGATTCAGGTGCCC-3′). The *Actin8* transcript was used as a constitutive control. PCR conditions were as follows: 32 cycles for *AtNUDX9*, and 20 cycles for *Actin8*. Thermal control for amplification was defined by cycles of 95 °C for 30 s, 55 °C for 30 s, and 72 °C for 60 s; and a final extension at 72 °C for 10min. The PCR products were analysed on 2% agarose gels. The equal loading of each amplified gene sequence was determined with the control *Actin8* PCR product.

### Western blot analysis

Western blot analysis was carried out as described previously by Yoshimura *et al.* (2004). Protein bands were detected by an anti-AtNUDX9 polyclonal rabbit antibody prepared using the recombinant protein as the primary antibody and an anti-rabbit IgG-horseradish peroxidase conjugate (Bio-Rad) as the secondary antibody.

### Plant materials and growth conditions

The mutants used in this study were derived from the wild-type *Arabidopsis* Col-0 ecotype. The knockout (KO)-*nudx9* (SALK_025038C), knockdown (KD)-*nudx9* (SALK_027992), and *vtc1-1* (CS8326) mutants were obtained from the ABRC. These plants were selfed to check for segregation and to obtain a purely homozygous line. T_3_ or M_3_ seeds were harvested and used for the experiments. Surface-sterilized wild-type and mutant seeds were sown on half-strength Murashige and Skoog (MS) medium containing 1% sucrose. Plates were stratified in darkness for 2 d at 4 °C and then transferred to a growth chamber kept at 23 °C during 16h of light (100 μmol photons m^–2^ s^–1^) and at 22 °C during 8h of darkness (normal growth conditions). In the assay for the NH_4_^+^ response, surface-sterilized seeds were grown on full-strength MS medium with or without ammonium nitrate (20.6mM)(but still containing 18.8mM potassium nitrate), and grown under normal growth conditions. The seedlings were collected as samples 4h after illumination. All experiments were repeated at least three times using independent batches of plant (more than 20 seedlings) as biological replicates.

### Leaf area determination

Total rosette surface area (hereafter called leaf area) was measured using Image J as described previously (Schindelin *et al.*, 2012; Maruta *et al.*, 2014).

### Measurement of AsA and DHA levels

Plant tissue was frozen in liquid N_2_ and used in the AsA and DHA analyses. AsA and DHA levels were determined spectrophotometrically using AsA oxidase as described previously (Maruta *et al.*, 2008).

### N-Glycoprotein analysis

Total protein samples were prepared as described above. Proteins in the supernatant were separated by SDS-PAGE in a 12% slab gel and transferred to polyvinylidene difluoride (PVDF) membranes (Bio-Rad, Hercules, CA, USA) using an electroblot apparatus (model 200/2.0, Bio-Rad). The membrane was incubated for 1h in PBSCT buffer (138mM NaCl, 2.7mM KCl, 10mM Na_2_HPO_4_, 1.8mM KH_2_PO_4_, 1mM MgCl_2_, 1mM CaCl_2_, 0.5% Tween 20, pH 7.2) and then for 1h in PBSCT buffer containing 0.1 μg ml^–1^ ConA conjugated to horseradish peroxidase (Sigma, St Louis, MO, USA). The membrane was washed five times for 5min with PBSCT. ConA-binding glycoproteins were detected with the ECL Select Western Blotting Detection System (GE Healthcare). The ConA-specific bands were quantified using Image J software.

### Statistical analyses

Statistical differences were evaluated by analysis of variance (ANOVA) using Microsoft Excel 2010 software (ver. 14.0). Differences were considered significant at a probability level of *P* <0.05.

### Accession numbers

*Arabidopsis* Genome Initiative locus identifiers for the genes described in this study are as follows: *AtNUDX1* (At1g68760), *AtNUDX2* (At5g47650), *AtNUDX3* (At1g79690), *AtNUDX4* (At1g18300), *AtNUDX5* (At2g04430), *AtNUDX6* (At2g04450), *AtNUDX7* (At4g12720), *AtNUDX8* (At5g47240), *AtNUDX9* (At3g46200), *AtNUDX10* (At4g25434), *AtNUDX11* (At5g45940), *AtNUDX25* (At1g30110), *VTC1* (At2g39770), *Actin8* (At1g49240).

## Results and discussion

### Identification of AtNUDXs having pyrophosphohydrolase activity toward GDP-d-Man

**Fig. 1.**
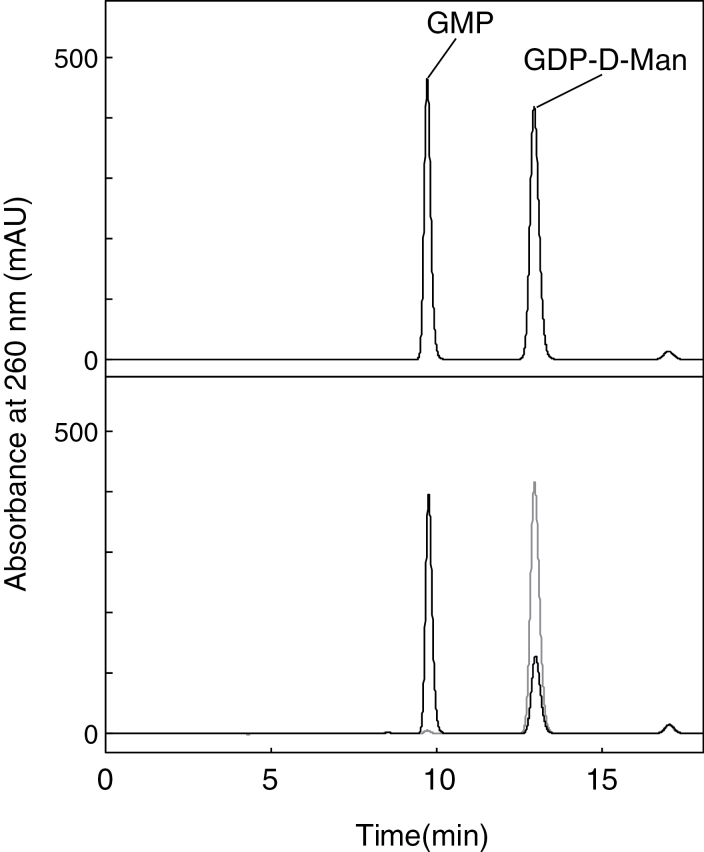
Identification of GDP-d-Man degradation products by AtNUDX9. Reaction mixture containing 100 μM (2 nmol per 20 μl) GDP-d-Man was incubated with 5mM Mg^2+^ in the absence or presence of the purified recombinant AtNUDX9 protein at 37 °C for 10min and then subjected to HPLC with the COSMOSIL C18 column as described in the Materials and methods. The elution profile of the reaction mixture without the enzyme (grey line) or with the enzyme (black line) is shown. Positions of standards (2 nmol of GMP and 2 nmol of GDP-d-Man) are shown in the upper panel.

Considering the subcellular localization of the enzymes involved in the synthesis of GDP-d-Man (Wheeler *et al.*, 1998; Conklin *et al.*, 1999), the AtNUDX(s) having activity toward GDP-d-Man should exist in the cytosol. Thus, the purified recombinant proteins of cytosolic-type AtNUDXs (AtNUDX1–11, 25) were prepared. Their pyrophosphohydrolase activities toward GDP-d-Man in the presence of 5mM Mg^2+^ as a cofactor were examined by HPLC analysis as described above. No activity could be detected in recombinant AtNUDX1-8, 10, 11, and 25, but a reduction was observed in the peak of GDP-d-Man in the reaction mixture containing recombinant AtNUDX9 (Fig. 1). In addition to the peak in GDP-d-Man as a substrate, the first peak that was eluted at approximately 9.7min corresponded to that of standard GMP. The activities were linear with time and amount of the enzyme, AtNUDX9. *E. coli* Orf191 has been shown to hydrolyse GDP-d-Man to GDP and mannose, whereas *E. coli* Orf1.9 hydrolyses GDP-d-Man to GMP and d-Man 1-phosphate (Frick *et al.*, 1995). Our results indicated that, similar to *E. coli* Orf1.9, AtNUDX9 exhibited pyrophosphohydrolase activity toward the diphosphate linkage in GDP-d-Man, and generated GMP and d-Man 1-phosphate as final products (see Supplementary Fig. S1at *JXB* online; Fig. 1). The possibility cannot be excluded that other members of the AtNUDX family have the GDP-d-Man pyrophosphohydrolases activity in the presence of other divalent cations, such as Mn^2+^ and Zn^2+^, as a cofactor. Ogawa *et al.* (2005) have already demonstrated that AtNUDX9 had no detectable activity toward various types of nucleotide sugars (ADP-d-ribose, ADP- and UDP-d-glucose, UDP-l-galactose), in addition to deoxynucleoside triphosphates (dNTPs), 8-oxo-7,8-dihydro- 2′-(deoxy) guanosine 5′-triphosphate, Ap_n_A, NADH, FAD, and coenzyme A. On the other hand, *E. coli* Orf1.9 was reported to have activity toward not only GDP-d-Man, but also GDP-d-glucose and GDP-l-fucose with relatively low efficiency (Frick *et al.*, 1995). However, AtNUDX9 had no detectable activity toward GDP-d-glucose and GDP-l-fucose. Therefore, it was concluded that AtNUDX9 has high specificity to GDP-d-Man as a substrate for its hydrolysis activity. This substrate and the resulting products (GDP-d-Man+H_2_O and d-Man 1-phosphate+GMP) corresponded partly to those of the reverse reaction of VTC1, which catalyses the reversible reaction (d-Man 1-phosphate+GTP↔GDP-d-Man+pyrophosphate) (Conklin *et al.*, 1999) (see Supplementary Fig. S1 at *JXB* online). It is possible that the AtNUDX9 reaction competes with that of VTC1 and perturbs the equilibration between intracellular d-Man 1-phosphate and the GDP-d-Man levels maintained by VTC1.

### Enzymatic properties and kinetic parameters of AtNUDX9 as a GDP-d-Man pyrophosphohydrolase

Divalent metal ions are required for the activity of Nudix hydrolases (Mildvan *et al.*, 2005). Therefore, the effects were examined of a series of divalent metal ions on the activity of AtNUDX9 (Table 1). No activity was detected in the absence of metal ions. The activity of AtNUDX9 was the highest in the presence of Mg^2+^ (5mM); it was 20–27 % in the presence of Zn^2+^, Mn^2+^, Ca^2+^, and Cu^2+^ (5mM each). The AtNUDX9 activity toward GDP-d-Man in the presence of 1mM Mg^2+^ was approximately 17% of that in 5mM Mg^2+^ (Table 1). Mg^2+^ is known most effectively to induce the activities of almost all of the other Nudix hydrolases from various organisms (Gasmi and McLennan, 2001; Okuda *et al.*, 2004; Klaus *et al.*, 2005; Ogawa *et al.*, 2005; Xu *et al.*, 2006; Ito *et al.*, 2012b; Goyer *et al.*, 2013). Considering that the typical cytosolic concentration of Mg^2+^ in plants is higher than that of the other divalent ions (Klaus *et al.*, 2005), it is suggested that the divalent ion essential for AtNUDX9 activity is Mg^2+^.

**Table 1.** The requirement of divalent cations for AtNUDX9 activity The requirement of divalent cations for GDP-d-Man pyrophosphohydrolase activity was determined in the presence and the absence of divalent cations (5mM) at 37 °C for 10min, as described in the Materials and methods.

Metal ions	Concentration	Relative Activity (%) GDP-d-Mannose
Mg^2+^	5 mM	100
	1 mM	17.1
None		<1.0
Zn^2+^	5 mM	20.6
Mn^2+^	5 mM	27.2
Ca^2+^	5 mM	23.9
Cu^2+^	5 mM	21.6

The kinetic parameters for GDP-d-Man were measured in the presence of Mg^2+^ (Table 2). The apparent *K*_m_ and *V*_max_ values for GDP-d-Man of AtNUDX9 were estimated from Lineweaver–Burk plots (see Supplementary Fig. S3 at *JXB* online). The *K*_m_ and *V*_max_ values of AtNUDX9 were 376±24 μM and 1.6±0.15 μmol min^–1^ mg^–1^ protein, respectively. The *V*_max_ of AtNUDX9 was almost the same as that of *E. coli* orf1.9 (1.6±0.1 μmol min^–1^ mg^–1^ protein), but was markedly lower than that of *E. coli* Orf191 (207±9.2 μmol min^–1^ mg^–1^ protein) (Xu *et al.*, 2006). On the other hand, the *K*_m_ value of AtNUDX9 was approximately 50% lower than that of *E. coli* Orf191 (810±120 μM). Therefore, AtNUDX9 had moderate catalytic efficiency (*k*_cat_ */K*_m_) for GDP-d-Man, similar to *E. coli* orf1.9.

**Table 2.** Analysis of the enzymatic properties of AtNUDX9 The standard assay was used with concentrations of 50 μM to 2mM for GDP-d-Man at 37 °C with 5mM Mg^2+^, as described in the Materials and methods. Data are means of three independent determinations ±SD.

	*K*_m_ (mM)	*V*_max_ (μmol min^–1^ mg^–1^ protein)	*k*_cat_ (s^–1^)	*k*_cat_/*K*_m_ (s^–1^ M^–1^)
AtNUDX9	0.376±0.024	1.61±0.15	0.93±1.50	2.4×10^3^
*E. coli* orf1.9^*a*^	0.30±0.08	1.6±0.1	0.49±0.03	1.6×10^3^
*E. coli* orf191^*b*^	0.81±0.12	207±9.2	75±3.3	9.3×10^4^

^*a*^ Frick *et al.* (1995).

^*b*^ Xu *et al.* (2006).

Previous studies reported that the activities of PMI1 and l-galactose dehydrogenase, which is involved in AsA biosynthesis, were inhibited by AsA itself, suggesting the existence of a feedback control mechanism (Mieda *et al.*, 2004; Maruta *et al.*, 2008), although it is debatable whether such inhibition occurs *in vivo* (Linster and Clarke, 2008). For example, incubation with 5mM AsA was shown to inhibit 52% of the activity of PMI1, an enzyme involved in the GDP-d-Man/AsA biosynthetic pathway (Maruta *et al.*, 2008). The activity of AtNUDX9 remained largely unchanged even after incubation with 5mM AsA (see Supplementary Table S1 at *JXB* online). The activity of AtNUDX9 was only slightly inhibited by incubation with H_2_O_2_ (see Supplementary Table S1 at *JXB* online). These results suggest that the activity of AtNUDX9 is independent of feedback control by AsA and the cellular redox state.

### Comparison of amino acid sequences between AtNUDX9 and other GDP-d-Man pyrophosphohydrolases from various organisms

**Fig. 2.**
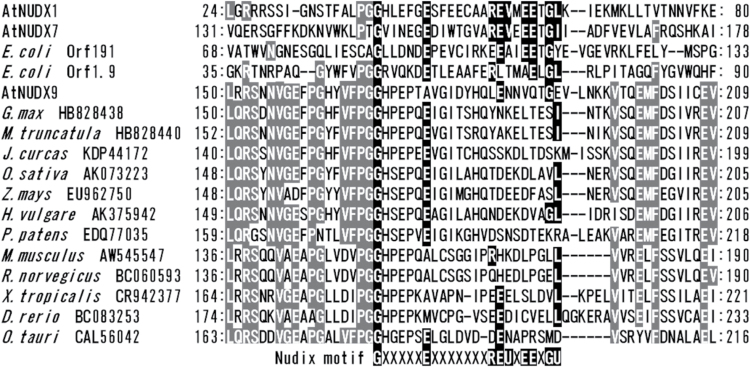
Alignment of partial amino acid sequences surrounding the Nudix motifs of AtNUDXs with other GDP-d-Mannose pyrophosphohydrolases from various organisms. Amino acids that were highly conserved are shown by grey boxes. Amino acids conserved in the Nudix motif G*X*_5_E*X*_7_REU*X*EE*X*GU (where U is a hydrophobic residue) are shown in black boxes. The sequences used here were as follows AtNUDX1, At1g68760; AtNUDX7, At4g12720; *Escherichia coli* Orf191, X77707; *Escherichia coli* Orf 1.9, L11721; AtNUDX9, At3g46200; *Glycine max* nudix hydrolase 9, HB828438; *Medicago truncatula* Nudix hydrolase, HB828440; *Jatropha curcas*, KDP44172; *Oryza sativa* hypothetical protein, AK073223; *Zea mays* nudix type motif 22, EU962750; *Hordeum vulgare* predicted protein, AK375942; *Physcomitrella patens* hypothetical protein, EDQ77035; *Mus musculus* Nudt22, AW545547; *Rattus norvegicus* Nudt22, BC060593; *Xenopus tropicalis* nudt22, CR942377; *Danio rerio* nudt22, BC083253; and *Ostreococcus tauri* hypothetical protein, CAL56042.

The amino acid sequences of AtNUDX9 were compared with other GDP-d-Man pyrophosphohydrolases from various organisms. As shown in Fig. 2, similar to AtNUDX1 and 7, the typical Nudix hydrolases contained the highly conserved Nudix motif as the active site. However, previous studies demonstrated that *E. coli* orf1.9, but not orf191, had amino-acid replacements in the Nudix motif, by which the conserved two glutamic acid residues and bulky aliphatic residue (isoleucine, valine, or leucine) were substituted by other residues, suggesting that such replacements were unique to the GDP-d-Man pyrophosphohydrolase (Frick *et al.*, 1995). Similar amino-acid replacements in the Nudix motif were also observed in the sequence of AtNUDX9, but not of the other AtNUDXs (Fig. 2), suggesting the involvement of such amino-acid replacements in the pyrophosphohydrolase activity toward the diphosphate linkage in GDP-d-Man. A conserved arginine, three glutamic acid residues, and two bulky aliphatic residues were substituted in the Nudix motif of AtNUDX9. On the other hand, AtNUDX9 showed only a slight identity (>14%) to both orf191 and orf1.9.

Nudix hydrolases containing the amino-acid replacements characteristic for the GDP-d-Man pyrophosphohydrolase from the National Center for Biotechnology Information (NCBI) database (http://www.ncbi.nlm.nih.gov/) were then examined. Similar to AtNUDX9, amino-acid replacements were observed in many putative Nudix hydrolases from dicots (*Glycine max* and *Medicago truncatula*), monocots (*Oryza sativa*, *Zea mays*, and *Hordeum vulgare*), bryophytes (*Physcomitrella patens*), green alga (*Ostreococcus tauri*), mammals (*Mus musculus* and *Rattus norvegicus*), amphibians (*Xenopus tropicalis*), and fish (*Danio rerio*) (Fig. 2). Those from dicots, monocots, bryophytes, and the green alga showed high identity (49–64%) to AtNUDX9, whereas those from mammals, amphibians, and fish were relatively low (28–31%). These results suggest that the GDP-d-Man pyrophosphohydrolase was derived from a common evolutionary origin and the enzymes from phototrophs have been developed separately from those of vertebrates.

### Effect of $NH_{4}{}^{+}$ on the AtNUDX9 expression and GDP-d-Man pyrophosphohydrolase activity

**Fig. 3.**
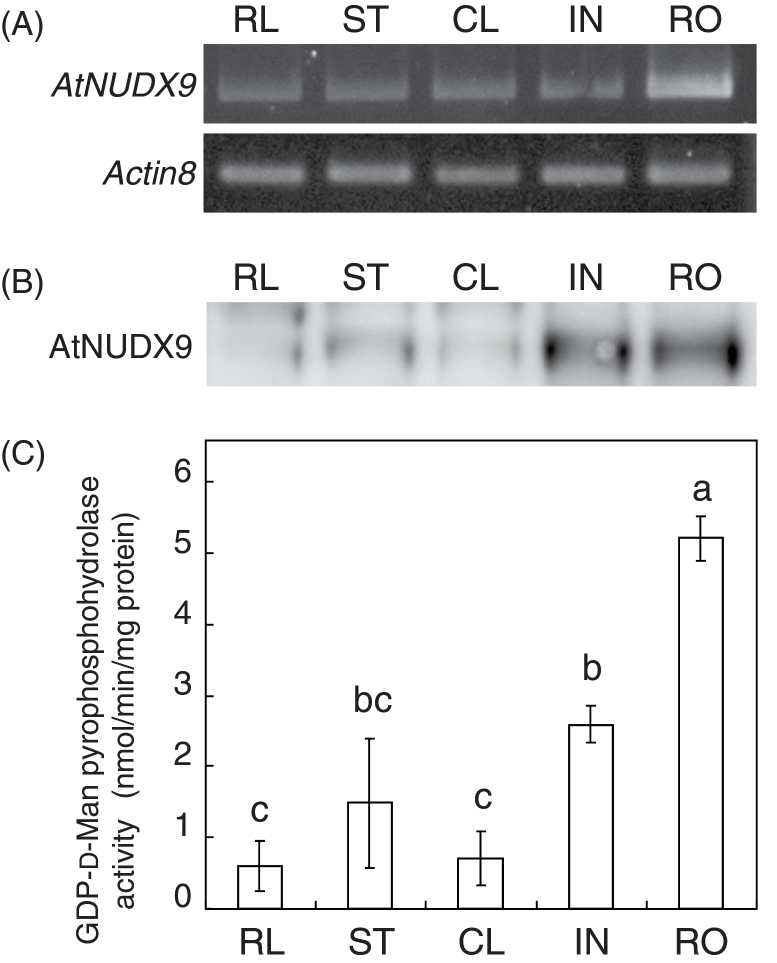
Expression of AtNUDX9 in various tissues of *Arabidopsis*. The mRNA and protein levels of AtNUDX9 and the activity of GDP-d-Man pyrophosphohydrolase in the rosette leaf (RL), stem (ST), root (RO), cauline leaf (CL), and inflorescence (IN) of 5-week-old *Arabidopsis* plants grown on half-strength MS medium were analysed. (A) Semi-quantitative RT-PCR analysis of AtNUDX9 mRNA. The equal loading of each amplified cDNA was determined with the control *Actin8* PCR product. (B) Western blot analysis of the AtNUDX9 protein. The AtNUDX9 protein was detected using a specific polyclonal antibody raised against the recombinant AtNUDX9 protein. (C) Total GDP-d-Man pyrophosphohydrolase activity. Data are means ±SD for three individual experiments (*n*=3) using plants grown independently. Details of the procedures used are described in the Materials and methods. Values without a common letter are significantly different according to ANOVA (*P* <0.05).

Changes in the expression levels of *AtNUDX9* were analysed in various tissues of 5-week-old wild-type *Arabidopsis* plants. Semi-quantitative RT-PCR analyses detected the expression of *AtNUDX9* in all tissues tested, including the rosette leaf, cauline leaf, stem, root, and inflorescence (Fig. 3). Among these tissues, the highest expression levels were detected in the roots. This result was inconsistent with previous findings (Ogawa *et al.*, 2005) and has been attributed to differences in the growth conditions used. As described below, the action of AtNUDX9 may be involved in response to ${\text{NH}}_{\text{4}}{}^{+}$ . In the present study, *Arabidopsis* plants were grown on a half-strength MS medium containing abundant ${\text{NH}}_{\text{4}}{}^{+}$ , whereas they were grown on soil in the previous study. A polyclonal antibody against the recombinant AtNUDX9 was produced to analyse protein levels. Western blot analyses showed that the AtNUDX9 antibody specifically cross-reacted with the recombinant AtNUDX9 protein (deduced molecular weights of AtNUDX9+hexahistidine-tag, 36.1kDa) (see Supplementary Fig. S4 at *JXB* online). Consistent with the results obtained by the semi-quantitative RT-PCR analysis, the levels of the AtNUDX9 protein (deduced molecular weights, 34.7kDa) were the highest in the roots, but were hardly detected in the rosette or cauline leaves (Fig. 3). The total activity of GDP-d-Man pyrophosphohydrolase in the extracts prepared from various tissues was assayed using the HPAEC-PAD system (Dionex). The results obtained showed that the levels of activities in respective tissues were similar to the levels of both AtNUDX9 mRNA and protein (Fig. 3). These results suggest that AtNUDX9 mainly acts in root tissues.

**Fig. 4.**
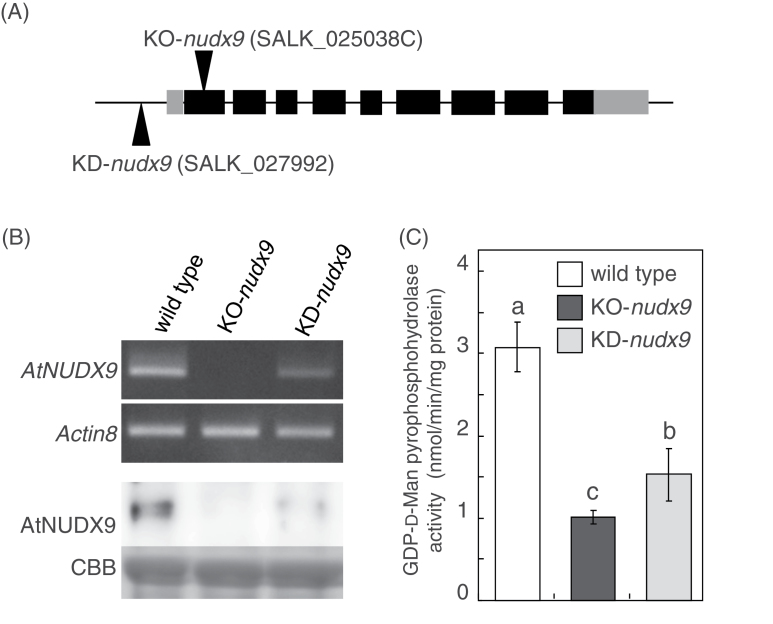
Characteristics of AtNUDX9-disrupted or -suppressed *Arabidopsis* plants. (A) Integration positions of T-DNA insertions in KO-*nudx9* and KD-*nudx9* plants. Exons and introns are represented by boxes and lines, respectively. The open reading frame and untranslated regions are indicated by black and grey boxes, respectively. (B) Semi-quantitative RT-PCR (top) and Western blot (bottom) analyses of the mRNA and protein levels, respectively, of AtNUDX9 in the rosette leaves of wild-type, KO-*nudx9*, and KD-*nudx9* plants. The plants were grown on half-strength MS medium for 2 weeks under normal growth conditions and then used for the analysis. Semi-quantitative RT-PCR analysis was performed using specific primers for *AtNUDX9* and *Actin8*. The equal loading of each amplified cDNA was determined with the control *Actin8* PCR product. The AtNUDX9 protein was detected by Western blotting using a specific polyclonal antibody raised against the recombinant AtNUDX9 protein. Coomassie Brilliant Blue (CBB) staining of protein gels was used to control for protein loading. (C) Total GDP-d-Man pyrophosphohydrolase activity in the leaves of wild-type, KO-*nudx9*, and KD-*nudx9* plants. Experimental conditions are the same as in (B). Data are means ±SD for three individual experiments (*n*=3) using plants grown independently. Details of the procedures used are described in the Materials and Methods. Values without a common letter were significantly different according to ANOVA (*P* <0.05).

In order to clarify the physiological function of AtNUDX9, two T-DNA insertion lines of AtNUDX9 (SALK_025038C; KO-*nudx9* and SALK_027992; KD-*nudx9*) were obtained. The T-DNA insertion sites of KO-*nudx9* and KD-*nudx9* are shown in Fig. 4A. Insertion resulted in the complete loss and a marked decrease in the expression of *AtNUDX9* in KO-*nudx9* plants and KD-*nudx9* plants, respectively (Fig. 4B). AtNUDX9 protein levels in the respective plants correlated well with the levels of mRNA (Fig. 4B). The activities of GDP-d-Man pyrophosphohydrolase in the KO-*nudx9* and KD-*nudx9* plants were 71% and 50% lower, respectively, than those in the wild-type plants (Fig. 4C). These results indicated that AtNUDX9 accounted for the majority of total GDP-d-Man pyrophosphohydrolase activity in *Arabidopsis* cells. Both plants grown on half-strength MS medium showed phenotypes similar to the wild-type plants under normal growth conditions (data not shown).

**Fig. 5.**
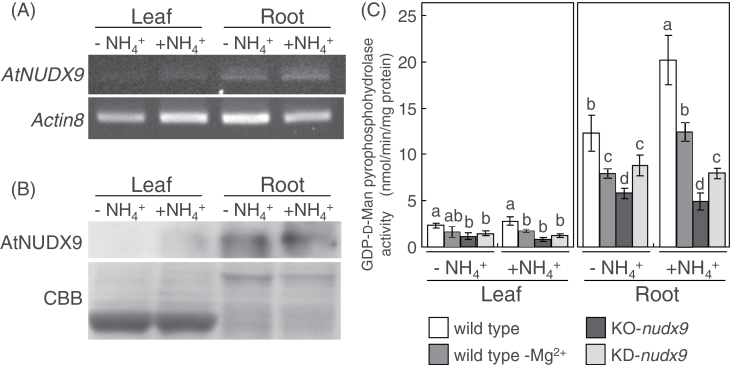
Expression of AtNUDX9 in response to $NH_{4}{}^{+}$ . The mRNA and protein levels of AtNUDX9 and the activity of GDP-d-Man pyrophosphohydrolase in the rosette leaf and root of *Arabidopsis* plants grown on full-strength MS medium in the presence (+$NH_{4}{}^{+}$ ) or absence (–$NH_{4}{}^{+}$ ) of ammonium nitrate for 10 d. (A) Semi-quantitative RT-PCR analysis of AtNUDX9 mRNA in the wild-type plants. The equal loading of each amplified cDNA was determined with the control *Actin8* PCR product. (B) Western blot analysis of the AtNUDX9 protein in the wild-type plants. The AtNUDX9 protein was detected using a specific polyclonal antibody raised against the recombinant AtNUDX9 protein. Coomassie Brilliant Blue (CBB) staining of protein gels was used to control for protein loading. (C) Total GDP-d-Man pyrophosphohydrolase activity in the wild-type, KO-*nudx9*, and KD-*nudx9* plants. The activity in the absence of Mg^2+^ in the reaction mixture was also shown (wild type –Mg^2+^). Data are means ±SD for three individual experiments (*n*=3) using plants grown independently. Details of the procedures used are described in the Materials and Methods. Within tissue, values without a common letter are significantly different according to ANOVA (*P* <0.05).

Barth *et al.* (2010) demonstrated that a mutation in *VTC1* resulted in enhanced sensitivity to ${\text{NH}}_{\text{4}}{}^{+}$ independent of intracellular AsA levels. Considering the catalytic reaction of VTC1, this result suggests that ${\text{NH}}_{\text{4}}{}^{+}$ sensitivity is facilitated by decreased levels of GDP-d-Man in plant cells. Qin *et al.* (2008) demonstrated that VTC1 activity was inhibited by the addition of ${\text{NH}}_{\text{4}}{}^{+}$ . Therefore, the levels of expression and activity of AtNUDX9 in wild-type *Arabidopsis* plants grown on full-strength MS medium in the presence (+${\text{NH}}_{\text{4}}{}^{+}$ , 20.6mM) or absence (–${\text{NH}}_{\text{4}}{}^{+}$ ) of ammonium nitrate were analysed. In both roots and leaves of 10-d-old wild-type plants, no difference was observed in the expression of AtNUDX9 between both media (Fig. 5A, B). However, it was found that the activity of GDP-d-Man pyrophosphohydrolase in the roots, but not in the leaves, was slightly, but significantly increased on the +${\text{NH}}_{\text{4}}{}^{+}$ medium (Fig. 5C), suggesting that the degradation of GDP-d-Man in the roots was activated in response to ${\text{NH}}_{\text{4}}{}^{+}$ . To clarify whether the increased activity under the +${\text{NH}}_{\text{4}}{}^{+}$ medium was dependent on AtNUDX9, Mg^2+^-dependent GDP-d-Man pyrophosphohydrolase activity was assayed. When Mg^2+^, as the cofactor, was not added to the reaction mixture, a slight and a marked decrease in the activities were observed in the leaf and roots, respectively, of wild-type plants grown on both –${\text{NH}}_{\text{4}}{}^{+}$ and +${\text{NH}}_{\text{4}}{}^{+}$ mediums compared with those in the presence of Mg^2+^ (Fig. 5C). Similarly, the activities in the leaves and roots of the KO-*nudx9* and KD-*nudx9* plants were significantly lower than those in the wild-type plants grown on the –${\text{NH}}_{\text{4}}{}^{+}$ and +${\text{NH}}_{\text{4}}{}^{+}$ media (Fig. 5C). However, the levels of activities in the KO plants were lower than those in the wild-type plant in the absence of Mg^2+^ (Fig. 5). The difference in the activities could be due to carried-over Mg^2+^ from the cells to the reaction mixture. The GDP-d-Man pyrophosphohydrolase activity of the KO-*nudx9* and KD-*nudx9* plants was not increased on the +${\text{NH}}_{\text{4}}{}^{+}$ medium. The activities of GDP-d-Man pyrophosphohydrolase in the leaf and root tissues of KO-*nudx9* plants grown on –${\text{NH}}_{\text{4}}{}^{+}$ medium were 51% and 53% lower, respectively, than those in the wild-type plants (Fig. 5C). On the other hand, the activities on +${\text{NH}}_{\text{4}}{}^{+}$ medium were 70% and 76% lower, respectively. These results indicated that AtNUDX9 accounted for 51% and 53% of the total GDP-d-Man pyrophosphohydrolase activity in the leaves and roots, respectively, under the absence of –${\text{NH}}_{\text{4}}{}^{+}$ and its contribution to the total activity was increased by 70% and 76% in the leaves and roots, respectively, in response to +${\text{NH}}_{\text{4}}{}^{+}$ . In addition, the AtNUDX9-dependent activities in the leaves and roots on +${\text{NH}}_{\text{4}}{}^{+}$ medium were increased by 1.7- and 2.4-fold, respectively, compared with those on –${\text{NH}}_{\text{4}}{}^{+}$ medium. On the other hand, the detection of the residual activities in the wild-type plants in the absence of Mg^2+^ and in the KO-*nudx9* plants suggests that, in addition to AtNUDX9, other enzyme(s) having the GDP-d-Man pyrophosphohydrolase activity occurs. The difference in the levels between mRNA and protein, and activity in the wild-type plants suggest that AtNUDX9 is activated in response to ${\text{NH}}_{\text{4}}{}^{+}$ possibly through a post-translational modification. However, the possibility could not be excluded that the semi-quantitative RT-PCR and Western blot analyses were less sensitive to detect the changes in expression levels of AtNUDX9 under different growth conditions. In addition, these findings, including the previous reports, suggest that the levels of GDP-d-Man are accurately regulated by the balance between the degradation and synthetic reactions, and the activation of AtNUDX9 and the inhibition of VTC1 by a high concentration of ${\text{NH}}_{\text{4}}{}^{+}$ cause a decrease in the levels of GDP-d-Man.

### AtNUDX9 is involved in $NH_{4}{}^{+}$ response

**Fig. 6.**
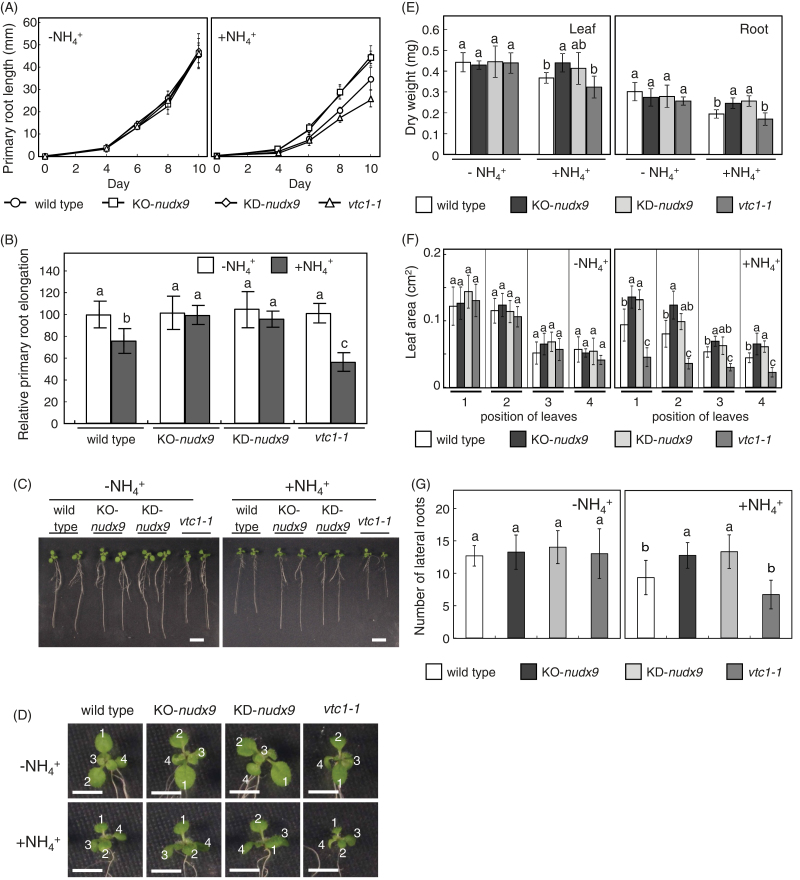
Ammonium sensitivity of AtNUDX9-disrupted or -suppressed *Arabidopsis* plants. Experimental conditions are the same as in Fig. 5. (A, B) Primary root length and relative root elongation, respectively, of the wild-type, KO-*nudx9*, KD-*nudx9*, and *vtc1-1* plants grown on +$NH_{4}{}^{+}$ and –$NH_{4}{}^{+}$ mediums. In the data of relative root elongation, 100% corresponds to a primary root length of respective plants grown on –$NH_{4}{}^{+}$ medium for 10 d. (C, D) Phenotypes. Scale bars, 1 and 0.5cm, respectively. The numbers (1 to 4) indicate the position of leaves. (E, F, G) Dry weight of leaves and roots, leaf area, and numbers of lateral roots of 10-d-old plants. The positions of leaves are described as in (D). Data represent means ±SD of 9–15 replicates. Values without a common letter were significantly different according to ANOVA (*P* <0.05). (This figure is available in colour at *JXB* online.)

In order to determine whether AtNUDX9 was involved in ${\text{NH}}_{\text{4}}{}^{+}$ sensitivity, the growth of wild-type, KO-*nudx9*, and KD-*nudx9* plants were compared as well as the *vtc1-1* mutants on the –${\text{NH}}_{\text{4}}{}^{+}$ and +${\text{NH}}_{\text{4}}{}^{+}$ media. Consistent with previous findings, root growth in the *vtc1-1* mutants was inhibited more than that by the wild-type plants on the +${\text{NH}}_{\text{4}}{}^{+}$ medium (Qin *et al.*, 2008; Barth *et al.*, 2010) (Fig. 6A–C). No significant difference was noted in primary root length between the wild-type plants and *vtc1-1* mutants on the –${\text{NH}}_{\text{4}}{}^{+}$ medium. An inverse correlation was observed between AtNUDX9 and VTC1 with regard to the effects of ${\text{NH}}_{\text{4}}{}^{+}$ on root growth. The primary root lengths of the KO-*nudx9* and KD-*nudx9* plants were longer than those of the wild-type plants on the +${\text{NH}}_{\text{4}}{}^{+}$ medium, but not on the –${\text{NH}}_{\text{4}}{}^{+}$ medium (Fig. 6A, C). The relative primary root elongation of KO-*nudx9* and KD-*nudx9* was sustained even under the +${\text{NH}}_{\text{4}}{}^{+}$ medium (Fig. 6B). Furthermore, the dry weight of roots and numbers of lateral roots of the KO-*nudx9* and KD-*nudx9* plants on the +${\text{NH}}_{\text{4}}{}^{+}$ medium were increased compared with those of the wild-type plants (Fig. 6E, G). These results indicated that AtNUDX9 negatively modulated root growth in response to ${\text{NH}}_{\text{4}}{}^{+}$ , possibly through the hydrolysis of GDP-d-Man. The aerial parts of all the genotypes tested here showed similar phenotypes on the –${\text{NH}}_{\text{4}}{}^{+}$ medium (Fig. 6C, D). On the other hand, the aerial parts of KO-*nudx9* and KD-*nudx9* plants were larger than the wild-type and *vtc1-1* plants on the +${\text{NH}}_{\text{4}}{}^{+}$ medium. In fact, the leaf size and dry weight of aerial parts of KO-*nudx9* and KD-*nudx9* plants on the +${\text{NH}}_{\text{4}}{}^{+}$ medium were significantly increased compared with those of the wild-type plants (Fig. 6E, F). Considering the high expression levels of AtNUDX9 in root tissues, it is likely that the enhanced root growth in the KO-*nudx9* and KD-*nudx9* plants causes facilitation of the growth of aerial parts.

### AtNUDX9 mutants have increased protein N-glycosylation

**Fig. 7.**
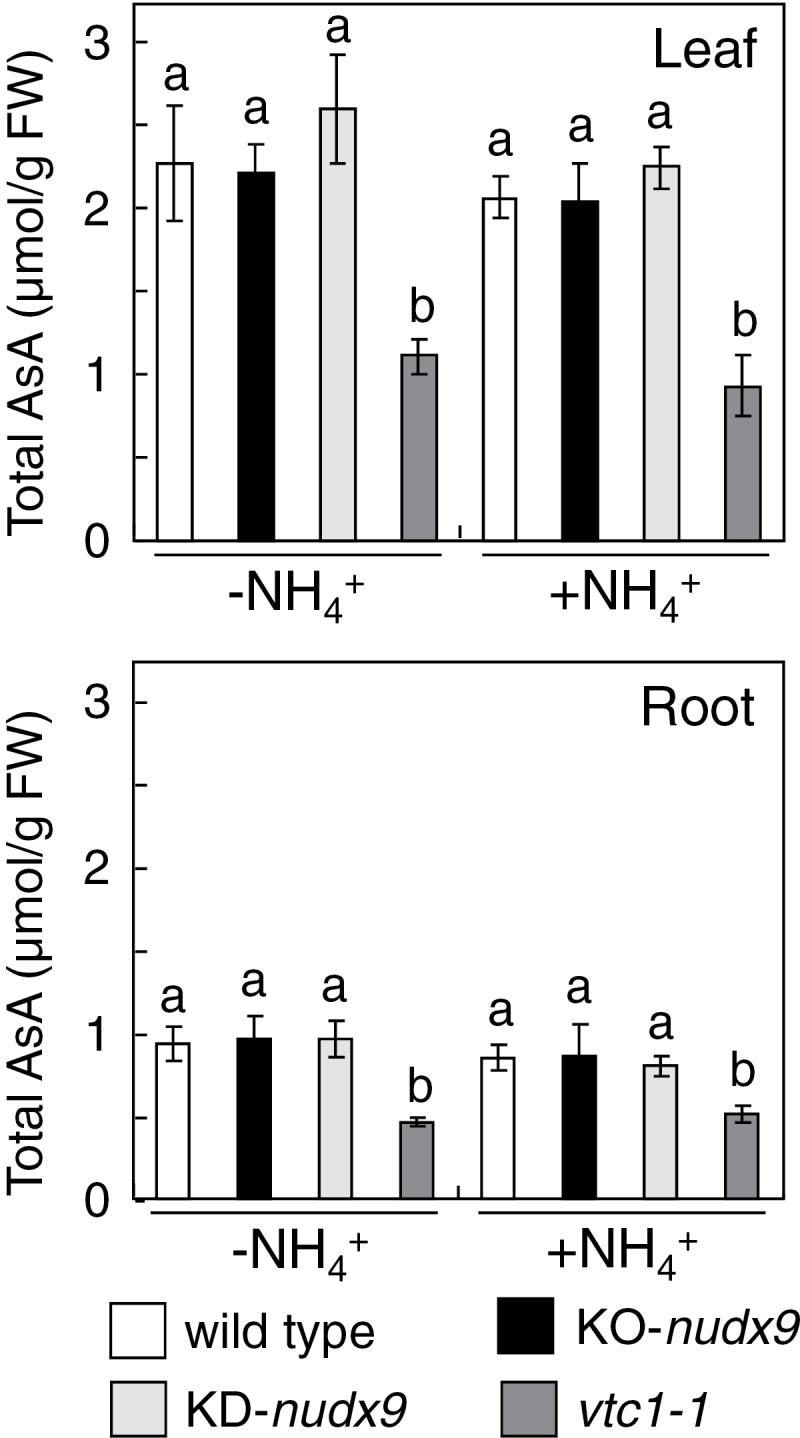
Changes in the levels of AsA in AtNUDX9-disrupted or -suppressed *Arabidopsis* plants in response to $NH_{4}{}^{+}$ . Experimental conditions are the same as in Fig. 5. The levels of AsA in the wild-type, KO-*nudx9*, KD-*nudx9*, and *vtc1-1* plants were analysed. Data are means±SD for three individual experiments (*n*=3) using plants grown independently. Details of the procedures used are described in the Materials and methods. Values without a common letter were significantly different according to ANOVA (*P* <0.05).

**Fig. 8.**
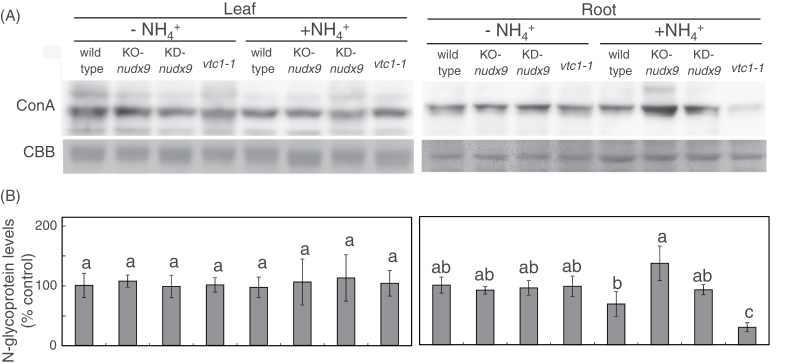
Changes in the levels of N-glycoprotein in AtNUDX9-disrupted or -suppressed *Arabidopsis* plants in response to $NH_{4}{}^{+}$ . Experimental conditions are the same as in Fig. 5. (A) The N-glycosylation of proteins in the wild-type, KO-*nudx9*, KD-*nudx9*, and *vtc1-1* plants was evaluated using a ConA-peroxidase reagent (top). Coomassie Brilliant Blue (CBB) staining of protein gels was used to control for protein loading (bottom). The same results were obtained in three independent experiments and the photograph showed representative result. (B) Quantification of the levels of protein N-glycosylation. The ConA-specific bands were quantified using Image J software. Values without a common letter were significantly different according to ANOVA (*P* <0.05).

The levels of AsA in the leaf and root tissues of the wild-type, KO-*nudx9*, KD-*nudx9*, and *vtc1-1* plants were compared in order to determine whether AtNUDX9 was involved in the regulation of the AsA biosynthesis. Consistent with previous findings, the levels of AsA in the leaf and root tissues of the *vtc1-1* mutants was significantly lower than those of wild-type plants grown on both –${\text{NH}}_{\text{4}}{}^{+}$ and +${\text{NH}}_{\text{4}}{}^{+}$ mediums (Qin *et al.*, 2008; Barth *et al.*, 2010) (Fig. 7). On the other hand, no significant difference was observed in the levels of AsA in the leaf and root tissues of the wild-type, KO-*nudx9*, and KD-*nudx9* plants grown on both mediums. These results suggest that the action of AtNUDX9 might be independent of the regulation of AsA biosynthesis, under at least normal growth conditions. The ${\text{NH}}_{\text{4}}{}^{+}$ -sensitive phenotype in the *vtc1-1* mutants has been attributed to N-glycosylation defects, indicating the importance of accurate levels of GDP-d-Man in N-glycosylation in root tissues of plants under a high concentration of ${\text{NH}}_{\text{4}}{}^{+}$ (Barth *et al.*, 2010). To investigate whether AtNUDX9 was involved in the N-glycosylation of some proteins, N-glycoprotein levels in the respective plants were compared using a peroxidase-conjugated ConA reagent, which bound to the branches of oligomannose chains on N-glycoproteins (Keller *et al.*, 1999). Levels of N-glycoprotein in the root tissues of the KO-*nudx9* and KD-*nudx9* plants were markedly and slightly higher, respectively, than those in the wild-type plants grown on the +${\text{NH}}_{\text{4}}{}^{+}$ medium, but not on the –${\text{NH}}_{\text{4}}{}^{+}$ medium, although the levels were low in the *vtc1-1* mutants (Fig. 8). On the other hand, no marked difference was observed in N-glycoprotein levels in the leaf tissues of the all genotypes grown on both mediums. These results indicated that increase and decrease of the levels of GDP-d-Man induced and suppressed, respectively, the rate of protein N-glycosylation in response to ${\text{NH}}_{\text{4}}{}^{+}$ and, therefore, the AtNUDX9 might prevent excessive protein N-glycosylation in response to ${\text{NH}}_{\text{4}}{}^{+}$ in the roots through the hydrolysis of GDP-d-Man. The *vtc1-1* mutant showed hyperstimulation of ${\text{NH}}_{\text{4}}{}^{+}$ efflux in the elongation zone, which was coincident with the ${\text{NH}}_{\text{4}}{}^{+}$ -mediated inhibition of root elongation (Li *et al.*, 2010), although the levels of ${\text{NH}}_{\text{4}}{}^{+}$ in the mutants were similar to those in the wild-type plants (Qin *et al.*, 2008; Barth *et al.*, 2010). This implied that high levels of GDP-d-Man suppressed ${\text{NH}}_{\text{4}}{}^{+}$ efflux and thereby enhanced tolerance to ${\text{NH}}_{\text{4}}{}^{+}$ in the KO-*nudx9* and KD-*nudx9* plants, possibly through the facilitation of protein N-glycosylation.

In the presence and absence of ${\text{NH}}_{\text{4}}{}^{+}$ , the *vtc1* mutation was reported to cause AsA deficiency in roots and leaves, while the mutation suppressed the levels of N-glycoprotein only in the roots on the +${\text{NH}}_{\text{4}}{}^{+}$ medium (Figs 7, 8), which was consistent with previous reports (Qin *et al.*, 2008). In addition, unlike the protein N-glycosylation, the AsA levels were not changed by the lack of AtNUDX9. These findings indicated that GDP-d-Man is preferentially utilized for protein N-glycosylation rather than the AsA biosynthesis in both leaf and root tissues regardless of ammonium conditions. This was also supported by the previous findings that the reaction catalysed by VTC2, but not VTC1, was a rate-limiting step of the AsA biosynthesis in *Arabidopsis* (Laing *et al.*, 2004; Bulley *et al.*, 2009, 2012; Yoshimura *et al.*, 2014). Thus, AtNUDX9 and VTC1 are likely to fine-tune the GDP-d-Man levels to modulate protein N-glycosylation, but not AsA biosynthesis, in the roots under ${\text{NH}}_{\text{4}}{}^{+}$ -abundant conditions. Since the Dol-phosphate-Man synthase 1 (DPMS1), catalysing the transfer of Man from GDP-d-Man to Dol-phosphate-Man, the main carrier of the Man residue at the first step of protein N-glycosylation, locates on the cytoplasmic face of the endoplasmic reticulum (Helenius and Aebi, 2008, Jadid *et al.*, 2011), the partitioning of GDP-d-Man in the cytosol would be important for the regulation of AsA biosynthesis and protein N-glycosylation. As described above, GDP-d-Man is also important for the synthesis of cell wall polysaccharides. It will be interesting to clarify how AtNUDX9 and VTC1 are involved in the regulation of carbon partitioning between AsA, N-glycosylated proteins, and cell-wall polysaccharides.

## Conclusion

In plant cells, GDP-d-Man is an important sugar donor in the biosynthesis of AsA and non-cellulosic cell-wall polysaccharides as well as post-translational modifications. The GDP-d-Man pyrophosphohydrolase was identified for the first time in plants here. Our results demonstrate that AtNUDX9 plays a role in the ${\text{NH}}_{\text{4}}{}^{+}$ response through the fine-tuning of GDP-d-Man levels in combination with VTC1 in the cytosol. AtNUDX9 and VTC1 constitute a futile cycle and therefore kinetic parameters, such as the *K*_m_ and *V*_max_ values, of those enzymes would affect the equilibration of the cycle. Although the kinetic parameters of VTC1 have not been demonstrated yet, it is likely that the production capacity of GDP-d-Man by the action of VTC1 is much higher than its hydrolysis by AtNUDX9, at least under normal conditions. On the other hand, net production of GDP-d-Man would decrease by activation of AtNUDX9 in response to environmental ${\text{NH}}_{\text{4}}{}^{+}$ concentration. It was reported that the earliest cellular response to ammonium uptake is the alkalinization of the cytosol (Britto and Kronzucker, 2005) and the activity of recombinant VTC1 is reduced by alkaline pH (Qin *et al.*, 2008). To uncover the importance of the degradation of GDP-d-Man by AtNUDX9, there is progress towards analysing the effects of the knockout and over-expression of *AtNUDX9* on GDP-d-Man metabolism and responses to various growth conditions.

## Supplementary data

Supplementary data can be found at *JXB* online.

Supplementary Fig. S1. Biosynthetic pathway for GDP-d-Man in higher plants.

Supplementary Fig. S2. Purification of recombinant AtNUDX1–11 and 25.

SupplementaryFig. S3. Affinity of recombinant AtNUDX9 for GDP-d-Man.

Supplementary Fig. S4. Antibody titers to the AtNUDX9 protein using the anti-AtNUDX9 polyclonal antibody.

Supplementary Table S1. Effect of AsA and H_2_O_2_ on the AtNUDX9 activity.

Supplementary Data
