# Supplementary material for: Identification and characterization of Arabidopsis AtNUDX9 as a GDP-d-mannose pyrophosphohydrolase: its involvement in root growth inhibition in response to ammonium
Source: J Exp Bot. 2015 Jun 6;66(19):5797–808. doi: 10.1093/jxb/erv281 (PMC4566977; doi:10.1093/jxb/erv281)
Supplement: Supplementary Data [file supp_66_19_5797__index.html]

Identification and characterization of Arabidopsis AtNUDX9 as a GDP-d-mannose pyrophosphohydrolase: its involvement in root growth inhibition in response to ammonium — Identification and characterization of Arabidopsis AtNUDX9 as a GDP-d-mannose pyrophosphohydrolase: its involvement in root growth inhibition in response to ammonium — Supplementary Data 

# Identification and characterization of *Arabidopsis* AtNUDX9 as a GDP-d-mannose pyrophosphohydrolase: its involvement in root growth inhibition in response to ammonium

## Supplementary Data

Data files

- Supplementary Data - Supplementary Data
