# Supplementary material for: Identification and characterization of Arabidopsis AtNUDX9 as a GDP-d-mannose pyrophosphohydrolase: its involvement in root growth inhibition in response to ammonium
Source: J Exp Bot. 2015 Jun 6;66(19):5797–808. doi: 10.1093/jxb/erv281 (PMC4566977; doi:10.1093/jxb/erv281)
Supplement: Supplementary Data [file supp_erv281_jexbot149039_file001.pdf]

Identification and characterization of the first member of an Arabidopsis GDP-D-mannose pyrophosphohydrolase, AtNUDX9: its involvement in root growth inhibition in response to ammonium

Hiroyuki Tanaka, Takanori Maruta, Takahisa Ogawa, Noriaki Tanabe, Masahiro Tamoi, Kazuya Yoshimura, and Shigeru Shigeoka

Supplemental Files

Supplementary Figure S1. Biosynthetic pathway for GDP-D-Man in higher plants.

Schematic representation of the biosynthetic pathway of GDP-D-Man, including the enzymatic reactions of VTC1 and AtNUDX9, is indicated. GDP-D-Man serves as a precursor of AsA and is essential for GPI-anchoring, protein N-glycosylation, and the synthesis of various structural cell wall polysaccharides.

Compound abbreviations: AsA, L-ascorbic acid; GMP, guanosine monophosphate; GTP, guanosine triphosphate; Pi, inorganic phosphate; PPi, inorganic pyrophosphate;

Enzyme abbreviations: GGP, GDP-L-galactose phosphorylase; GME, GDP-D-mannose-3',5'-epimerase; GMP, GDP-D-mannose pyrophosphorylase; GPP, L-galactose 1-phosphate phosphatase; PMI, phosphomannose isomerase; PMM, phosphomannomutase.

Supplemental Figure S2 Purification of recombinant AtNUDX1-11 and 25.

Recombinant AtNUDX1-11 and 25 protein was overexpressed in *E. coli*, purified with Ni^{2+} affinity chromatography, and verified using SDS-PAGE (4-20% gradient) with Coomassie Blue staining. The experimental conditions are described in "Materials and Methods". One μg of the purified recombinant AtNUDX1-11 and 25 proteins were loaded. M, molecular mass standards; Lanes 1, AtNUDX1 (deduced molecular weight, 18.9 kDa); Lane 2, AtNUDX2 (34.1 kDa); Lane 3, AtNUDX3 (89.4 kDa); Lane 4, AtNUDX4 (25.7 kDa); Lane 5, AtNUDX5 (36.8 kDa); Lane 6, AtNUDX6 (34.5 kDa); Lane 7, AtNUDX7 (39.0 kDa); Lane 8, AtNUDX8 (89.3 kDa); Lane 9, AtNUDX9 (36.8 kDa); Lane 10, AtNUDX10 (33.9 kDa); Lane 11, AtNUDX11 (27.7 kDa); Lane 12, AtNUDX25 (21.9 kDa).

Supplemental Figure S3 Affinity of recombinant AtNUDX9 for GDP-D-Man.

Initial velocities of the GDP-D-Man pyrophosphohydrolase activity were assayed as described in "Materials and Methods" with varied concentrations of GDP-D-Man. The kinetic constants were calculated from the Lineweaver-Burk plots based on the best fit lines. The data represent mean values from three repetitive experiments.

Supplementary Figure S4. Antibody titers to the AtNUDX9 protein using the anti-AtNUDX9 polyclonal antibody.

The recombinant AtNUDX9 protein (5, 10, 25, 50, 250 ng, lanes 1 to 5) was recognized by the anti-AtNUDX9 polyclonal antibody in the western blot analysis.

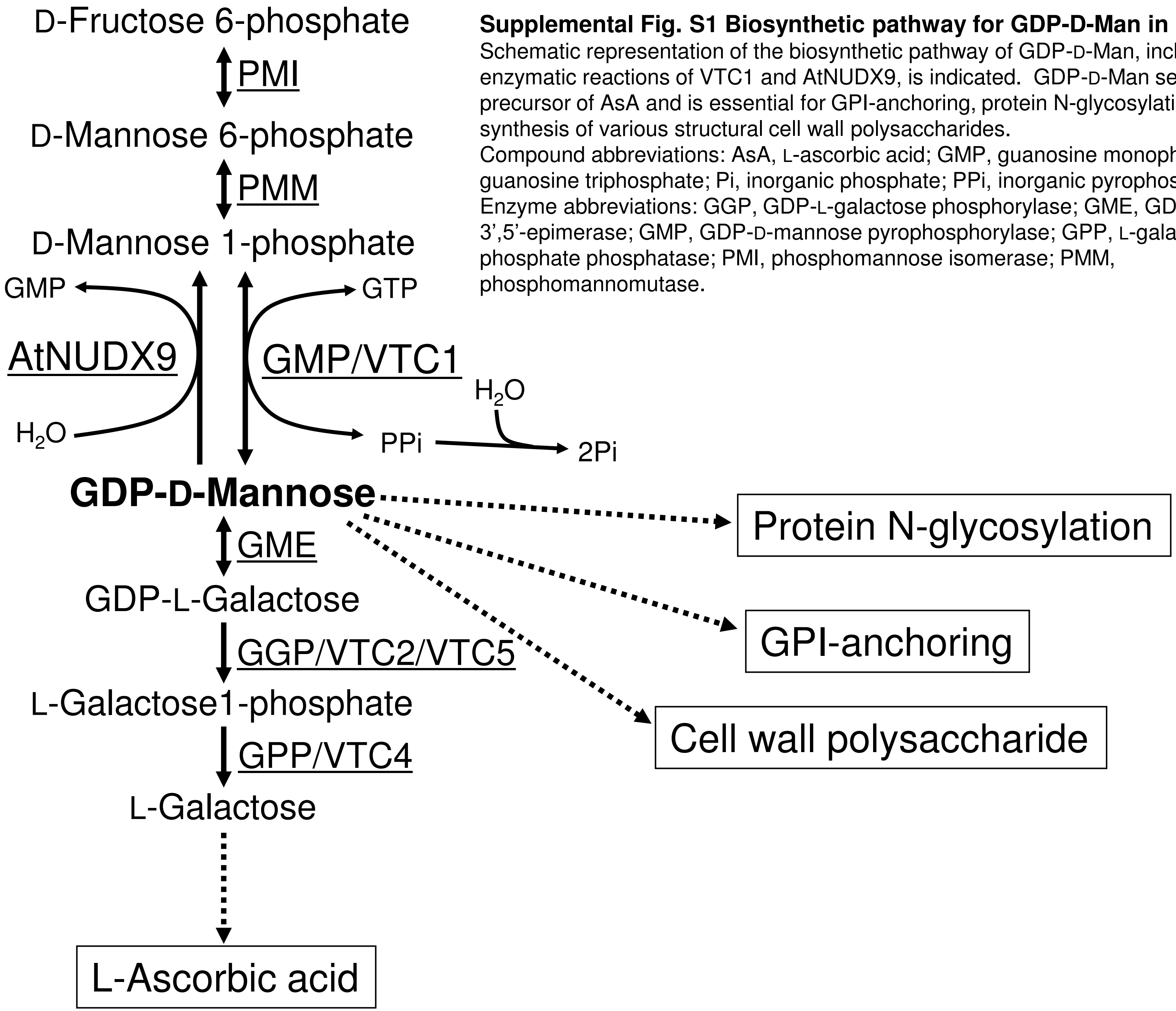

Supplemental Fig. S1 Biosynthetic pathway for GDP-D-Man in higher plants. Schematic representation of the biosynthetic pathway of GDP-D-Man, including the enzymatic reactions of VTC1 and AtNUDX9, is indicated. GDP-D-Man serves as a precursor of AsA and is essential for GPI-anchoring, protein N-glycosylation, and the synthesis of various structural cell wall polysaccharides.
Compound abbreviations: AsA, L-ascorbic acid; GMP, guanosine monophosphate; GTP, guanosine triphosphate; Pi, inorganic phosphate; PPi, inorganic pyrophosphate.
Enzyme abbreviations: GGP, GDP-L-galactose phosphorylase; GME, GDP-D-mannose-3’,5’-epimerase; GMP, GDP-D-mannose pyrophosphorylase; GPP, L-galactose 1-phosphate phosphatase; PMI, phosphomannose isomerase; PMM, phosphomannomutase.

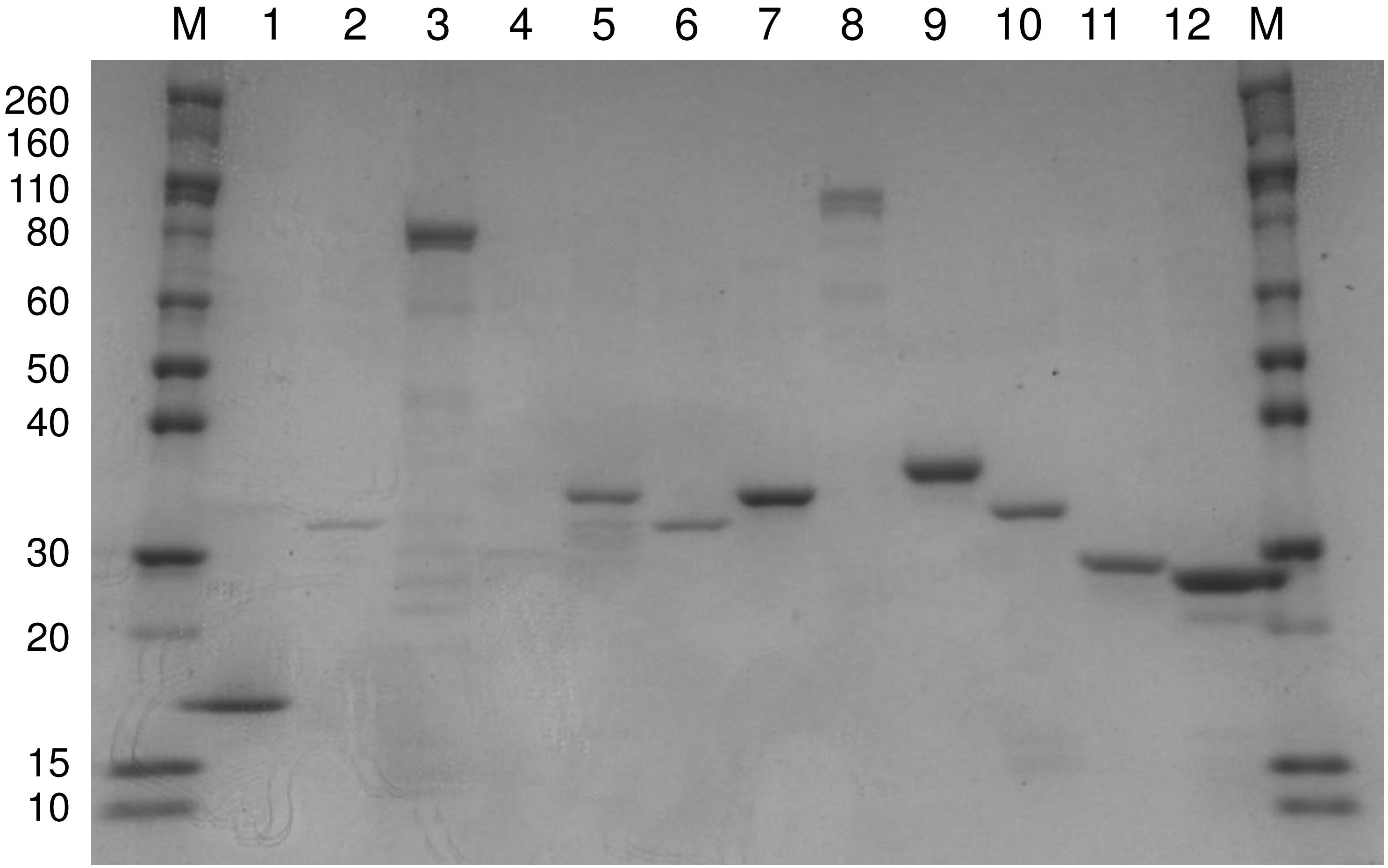

Supplemental Fig. S2 Purification of recombinant AtNUDX1-11 and 25.
Recombinant AtNUDX1-11 and 25 protein was overexpressed in *E. coli*, purified with Ni^{2+} affinity chromatography, and verified using SDS-PAGE (4-20% gradient) with Coomassie Blue staining. The experimental conditions are described in “Materials and Methods”. One µg of the purified recombinant AtNUDX1-11 and 25 proteins were loaded. M, molecular mass standards; Lanes 1, AtNUDX1 (18.9 kDa); Lanes 2, AtNUDX2 (34.1 kDa); Lanes 3, AtNUDX3 (89.4 kDa); Lanes 4, AtNUDX4 (25.7 kDa); Lanes 5, AtNUDX5 (36.8 kDa); Lanes 6, AtNUDX6 (34.5 kDa); Lanes 7, AtNUDX7 (39.0 kDa); Lanes 8, AtNUDX8 (89.3 kDa); Lanes 9, AtNUDX9 (36.8 kDa); Lanes 10, AtNUDX10 (33.9 kDa); Lanes 11, AtNUDX11 (27.7 kDa); Lanes 12, AtNUDX25 (21.9 kDa).

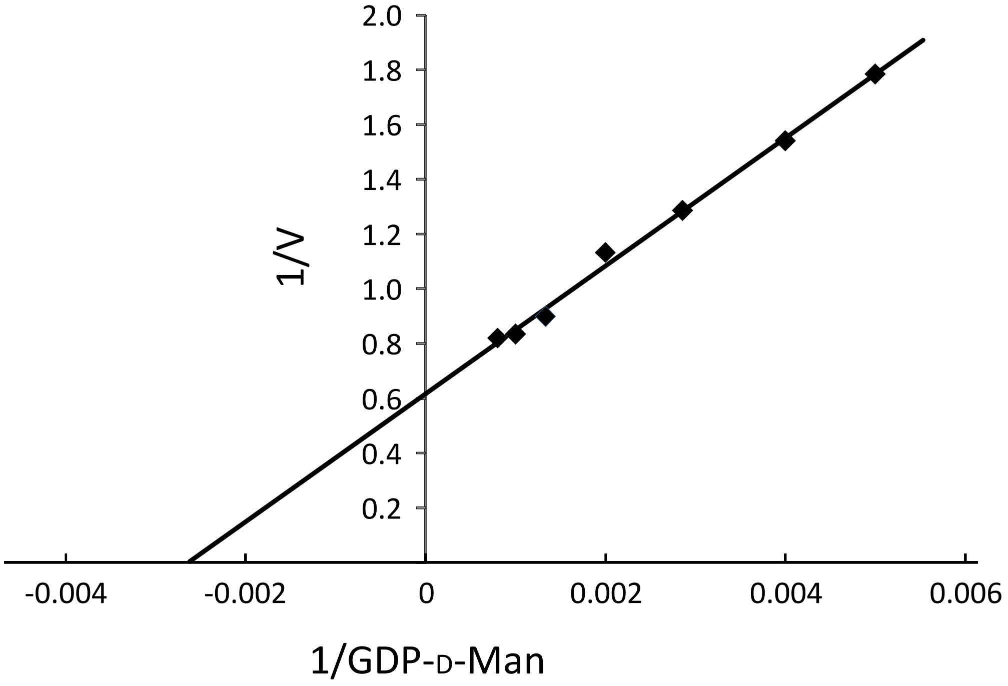

Supplemental Fig. S3 Affinity of recombinant AtNUDX9 for GDP-D-Man.
Initial velocities were assayed as described under ” Materials and Methods“ with varied concentrations of GDP-D-Man. The kinetic constants were calculated from the Lineweaver-Burk plots based on the best fit lines. The data represent mean values from three repetitive experiments.

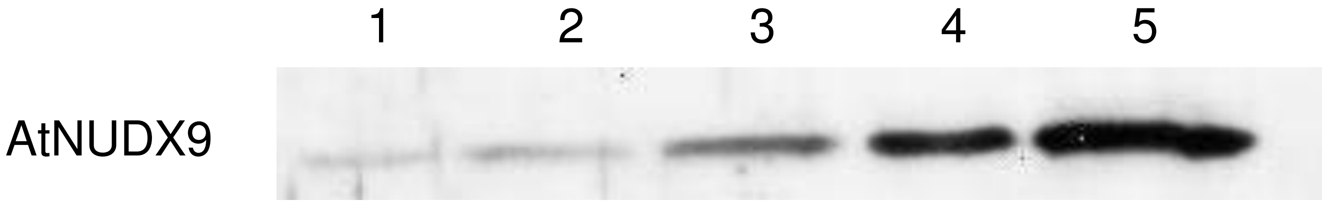

Supplemental Fig. S4 Antibody titers to the AtNUDX9 protein using the anti-AtNUDX9 porycloncal antibody.

The recombinant AtNUDX9 protein (5, 10, 25, 50, 250 ng, lanes 1 to 5) was recognized by the anti-AtNUDX9 polyclonal antibody in the western blot analysis.

Supplemental Table S1. Effect of AsA and H_2O_2 on the AtNUDX9 activity.

Each enzyme was incubated with various compounds for 15 min at 37 °C, and then the activity was measured as described under "Experimental Procedures."

**Compound**	**Concentration**	**Relative Activity (%)**	
		AtNUDX9	PMI[a]
None		100	100
AsA	1 mM	100	77
	5 mM	94	48
H_2O_2	1 mM	83	61
	5 mM	79	54

[a] Maruta et al. 2008
